# Supplementary material for: Incidence of shoulder dislocations in the UK, 1995–2015: a population-based cohort study
Source: BMJ Open. 2017 Nov 14;7(11):e016112. doi: 10.1136/bmjopen-2017-016112 (PMC5695490; doi:10.1136/bmjopen-2017-016112)
Supplement: Supplementary file 1 [file bmjopen-2017-016112supp001.pdf]

Supplementary table A: Clinical Practice Research Datalink (CPRD) dataset dislocation READ codes used to identify shoulder dislocation patients

**Description**	**CPRD READ Code**	**Numbers of patients**
Dislocation or subluxation of shoulder	**S41..00**	9,600
Dislocation of shoulder NOS	**S41z.00**	2,066
H/O: dislocated shoulder	**14G5.00**	2,331
Closed reduction of dislocation of shoulder	**7K6G300**	739
Closed traumatic dislocation of shoulder	**S410.00**	410
Recurrent dislocation of shoulder – anterior	**N083A00**	646
Anterior dislocation of shoulder	**S410111**	424
Recurrent joint dislocation, of shoulder region	**N083100**	176
Recurrent subluxation of shoulder – anterior	**N083C00**	168
Closed traumatic dislocation shoulder jnt. Anterior (sub-coracoid)	**S410100**	64
Closed traumatic dislocation shoulder joint, unspecified	**S410000**	78
Closed traumatic subluxation, shoulder	**S412.00**	61
**Total**		**16,763**
