# Supplementary figures and images for: Incidence of shoulder dislocations in the UK, 1995–2015: a population-based cohort study
Source: BMJ Open. 2017 Nov 14;7(11):e016112. doi: 10.1136/bmjopen-2017-016112 (PMC5695490; doi:10.1136/bmjopen-2017-016112)

## Validation Algorithm

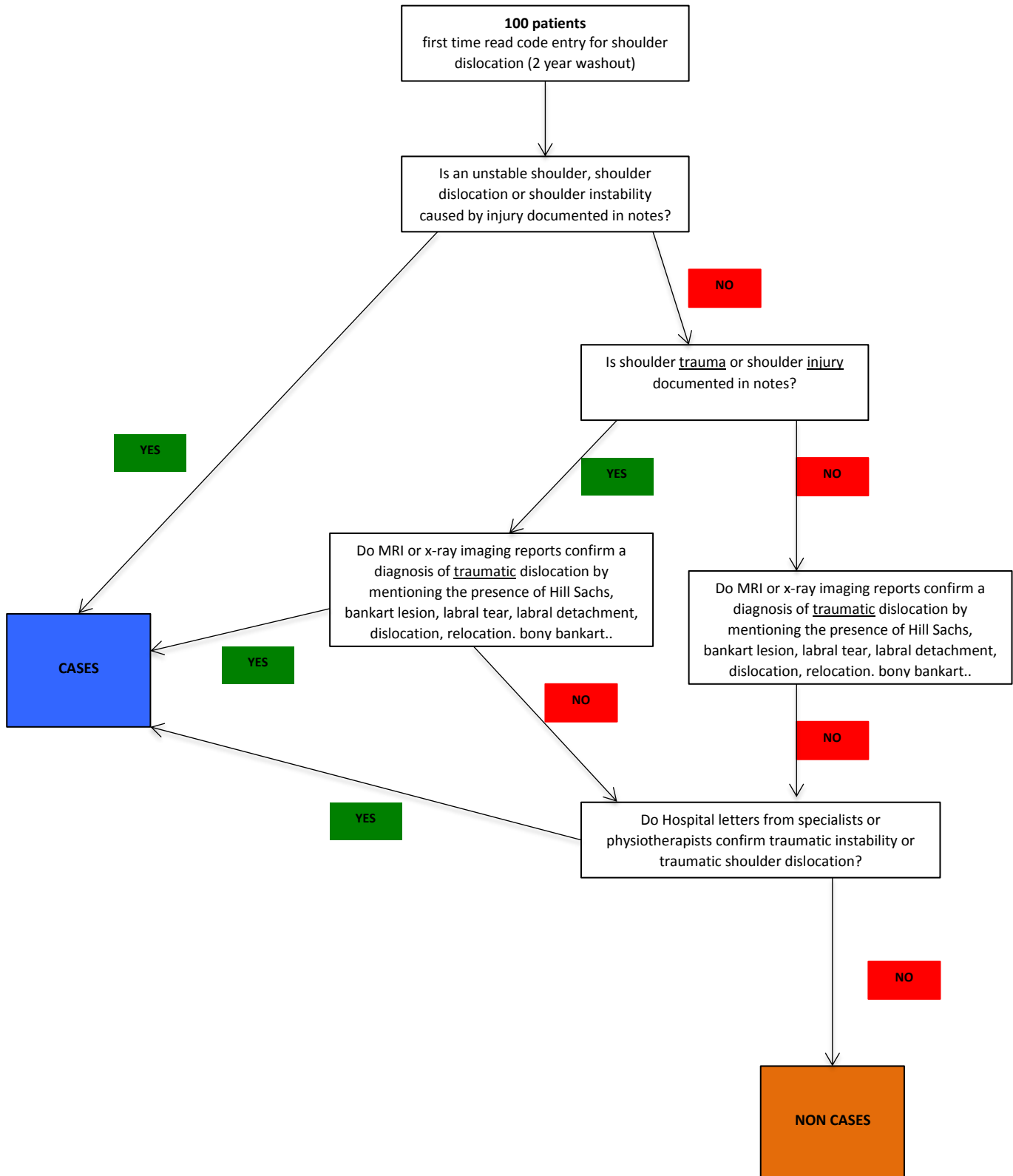

Supplement: Supplementary file 2 [file bmjopen-2017-016112supp002.pdf]
