# Supplementary material for: Incidence of shoulder dislocations in the UK, 1995–2015: a population-based cohort study
Source: BMJ Open. 2017 Nov 14;7(11):e016112. doi: 10.1136/bmjopen-2017-016112 (PMC5695490; doi:10.1136/bmjopen-2017-016112)
Supplement: Author's manuscript [file bmjopen-2017-016112.draft_revisions.pdf]

BMJ Open is committed to open peer review. As part of this commitment we make the peer review history of every article we publish publicly available.

When an article is published we post the peer reviewers' comments and the authors' responses online. We also post the versions of the paper that were used during peer review. These are the versions that the peer review comments apply to.

The versions of the paper that follow are the versions that were submitted during the peer review process. They are not the versions of record or the final published versions. They should not be cited or distributed as the published version of this manuscript.

BMJ Open is an open access journal and the full, final, typeset and author-corrected version of record of the manuscript is available on our site with no access controls, subscription charges or pay-per-view fees (http://bmjopen.bmj.com).

If you have any questions on BMJ Open's open peer review process please email editorial.bmjopen@bmj.com

BMJ Open

Incidence of shoulder dislocations in the United Kingdom, 1995-2015: a population-based cohort study

Journal:	*BMJ Open*
Manuscript ID	bmjopen-2017-016112
Article Type:	Research
Date Submitted by the Author:	30-Jan-2017
Complete List of Authors:	Shah, Anjali; University of Oxford, Nuffield Department of Orthopaedics, Rheumatology, and Musculoskeletal Sciences<br>Judge, Andrew; University of Oxford, NIHR Musculoskeletal Biomedical Research Unit<br>Delmestri, Antonella; University of Oxford, Nuffield Department of Orthopaedics, Rheumatology, and Musculoskeletal Sciences<br>Edwards, Katherine; University of Oxford, Nuffield Department of Orthopaedics, Rheumatology, and Musculoskeletal Sciences<br>Arden, Nigel; Nuffield Department of Orthopaedics, ;<br>Prieto-Alhambra, Daniel; University of Oxford<br>Holt, Tim; Oxford University, Department of Primary Care Health Sciences<br>Pinedo-Villanueva, Rafael; University of Oxford, Nuffield Department of Orthopaedics, Rheumatology, and Musculoskeletal Sciences<br>Hopewell, Sally; University of Oxford, Centre for Statistics in Medicine<br>Lamb, Sarah; University of Oxford,<br>Rangan, Amar; University of Oxford,<br>Carr, Andrew; University of Oxford, Nuffield Department of Orthopaedics, Rheumatology and Musculoskeletal Sciences<br>Collins, Gary; University of Oxford, Centre for Statistics in Medicine<br>Rees, Jonathan; University of Oxford,
**Primary Subject Heading**:	Epidemiology
Secondary Subject Heading:	Sports and exercise medicine
Keywords:	shoulder dislocation, incidence, Clinical Practice Research Datalink, CPRD

SCHOLARONE™ Manuscripts

ABSTRACT

Objective

/ & % 1 ' / # / / / #

' & / 2 4

Setting

2 ' & / # , / / / " / 1 A, " B

#& ' + ;> & / @@*

> *4 , # ' & / % , " -

C % / 4

Participants

/ # + ;+! / ' + ;> & @@* > *

4

Primary outcome measure

D / / ' >> >>> ' & @*E / # / %

/ / 4

Results

, / / # / , " ?@E A@*E ,D< ?!E @*EB

/ # / F' &G ;+E A@*E ,D< +;E ?*EB4

% & ' / # / // 4 % / /

(>4(' >> >>> ' & A@* E ,D< (>4((>4(B

For peer review only

4 ' >> >>> ' & A@*E ,D< *4* *4*B4

/ / % + > & A?>4 * ' >> >>> ' & H

@*E ,D< ?>4* ?>4+B4 D / / / ' 1 # ?4+ '

>> >>> ' & + ;> & 4

Conclusions

/ / # /

' & / # # #

2 % ' / 4 9 ' & / //

& 3' / # / / /

4 # 1 4 5 1 / &

D " 3 ' / # / / 4

Study registration< # & " % & D '

/ # / % & , AD , *I +>B # 0 / J /

' / " & /& A0 " B4

Strengths and limitation of this study

- # / / # /

  2 ' ' # ' & / 4

- # / / ' 1 #

  ?4+ ' >> >>> ' & + ;> &

- / # / % & / '

  / / - C 4

For peer review only

- % 3 / & / + !* & ' %
  & / # / ## #
  ' ' ;> & 4
- 9 - ' / / ' C
  ' & ' % ' &
  ' / / / / 4

For peer review only

INTRODUCTION

/ / / '

// /& ' / / @*E # /

/ 4 ! / /

/ & // # / & & ' ' &

/ ' / 8 1 (/ *4

/ F G / % '

?*E + @ E ; & ' ' 4 & / /

' / & ' & ' ' #

& # & / 4

' ' & / / & % 4 ? &

/ % 4;E # ' '

? ;> & / 4 D * & # ' & # '

(> & / / / & ' '

; E # ' & ## / / "

;E !> (> & 4 @ & ' / / #

/ & / ' ' & 14

> (D & # * ' * !* & ##

/ # / / A?+EB ' & / / ' 4 *

.# +>E ## ' / # /

/ // !4! 4

' / / # 4 +4 ' >> >>> '

& # / 4 + > D > > K / J . ? 3

For peer review only

/ / # / / ' # '

' # >> ' /& ' / 2

>> >>+ / / / D & % / & 4

% & ' / # / // / /

% > @ & A(;4? ' >> >>> ' & H @*E ,D< (4>

*(4*B4 % / / !(4@ ' >> >>> ' & A@*E

,D< !>4 !@4;B # !4! ' >> >>> ' & A@*E ,D< 4+

*4>B4

D > ( ) 3 4 % / / # ' &

/ ' + ;> & / / #

' >> L ' > > . , 4 % & # '

/ # / / /

% + > & A@?4! ' >> >>> ' & B4 %

/ / # ' & K / J

. 4 ?

/ / # ' & / 3

' 4 / / # / 2

1 ' ' 2 & ' % &

1 4

& % ' / # / / /

' & / / ' & / > & '

@@* > * 2 4

For peer review only

For peer review only

METHODS

Data source

' ' & / # , / / / " / 1

A, " B #& / # ' /

+ ;> & @@* > * 2 4 # & " % &

D ' / # / % & , AD , *I +>B # 0 / J

/ ' / " & /& A0 " B4

, " / % 4! ' ' # +;(2 ' / / /

' % / % # " 3 & +4@E # 2 ' '

& ' % # 2 ' ' # 3 / &4

9 & ' ' / / %

' / # ' % ' /

& 3 A80D 1 M B 1 A/ 1 3 1

1 B 1 A/ 1 3 1 1 B

% 4 D 3 # 0 ' ' % >>(! # '

1 , " 4 , / & / / /

' # , " " / 4 , " ' '

, " & % & 4

Participants

/ # " / # #

/ & ' / / / 3' / / / ' / /

' / / A " & B4 & ' &

For peer review only

/ / ' ' % /

/ & # / / A & ' #

- ' / / / # F ' G

' # - ' / / B ' / #

/ 4 ' # # 3/ / " , "

A5 B4

Validation of the coding of shoulder dislocations in CPRD

' % 3 / # / # /

, " / / ' + !* &

' @@; ! 0 / > * " / #

/ & ' 4 ' / # /

% 3 / & / % # ' &

/ '4 + !* &

& # / & / 1

' ' / # / ' 4

- C % A " &

8B / # - 4 ' # ; '

/ , " ' C ' G - ' / / #

/ / / ' & / ' / , " &

' G / / / 4 5 % & 1

- ' / / 4 # C &

/ ' / C % & / /

' / 4

For peer review only

% / ' # ' /

/ / , " <

B ' % ' / % % # // / /

, " # ;*E4

B ' % ' / % % # // F' & G F# G

/ / , " # ;*E4

Statistical analysis of the data

/ ' % / ' & # ' &

/ & ' / # / 4 D / / & ' >> >>>

' & / / @*E / # / % ' %

/ / % (4 4 (

RESULTS

Validation of the coding of shoulder dislocations within CPRD

' # ; ' + !* & , "

/ # # + >(+4 .# ; -

% C # @* A**EB ' 4 5 ' -

/ # ' # # ' / / / /

, " & % # 4 ' /

/ / / #< A B , " / + !* & ' @@; L !

0 / > * A N+ >(+B A B # ; ' & / %

For peer review only

C - # # A/B '

A B ' - C % 4

For peer review only

< ' / / / / # / ' + !*

& / , / / / " / 1 '

@@; ! 0 / > * ' ' , "

- % C

Demographic characteristic	Whole cohort*	All GP questionnaires*	Responders*	Non-responders*
, O	+ >(+	;	@;	;*
-				
0	(@@ A?!EB	!; A?>EB	? A?(EB	*+ A;*EB
5	>** A ;EB	!* A >EB	+ A +EB	@ A *EB
0 A& B J C	(A > !(B	(A > @B	(A > @B	(A @ @B
0 80D A1 M B J C	(A ;B	(A ;B	* A ?B	! A +B
0 , / & 3 J C	> A> >B	> A> >B	> A> >B	> A> >B
"				
0	+! A(EB	> A>EB	> A>EB	> A>EB
#	+;! A EB	A EB	; A ?EB	(A*EB
)	+@* A EB	! A !EB	A EB	A +EB
	!! A EB	(A EB	! A!EB	A EB
9	@* A +EB	@ A ;EB	! A !EB	+ A EB
,	@+* A +EB	! A @EB	; A ?EB	* A >EB
,	;> A EB	* A *EB	A EB	! A ;EB
9	;(! A EB	; A >EB	; A ?EB	> A>EB
9 0	++; A EB	* A@EB	; A;EB	? A EB
6 1 J	*(A(EB	+ A!EB	> A>EB	+ A?EB
D 3 # 0 ' ' % >>(				
##	;@ A EB	*! A! EB	? A @EB	+ A!*EB
	>;; A ?EB	!* A >EB	> A EB	* A >EB
!	@*? A +EB	(A (EB	+ A +EB	? A EB
(	?;+ A (EB	!? A EB	@ A >EB	? A (EB
' %	+ (A >EB	A !EB	(A (EB	? A EB
0	! A >EB	> A>EB	> A>EB	> A>EB

For Peer review only

7 ' / AEB ' 4

, / / / 80D , / & /

/ ' 4 >>E ' / % # - ' / /

9 # ' # / #

' / 4 C & 1 D 3 # 0 '

' % % ' ' # ' / A ## B

(A ' % B ' ' ' # + >(+

' ' # ' % ' 4

/ / # % / / / & , " #

?@E A@*E ,D< ?!E @*EB # ' A B4 - / #

E A > ' B / ' ##

/ / ' / #

/ / % / 4 F' &G # / / #

;+E A@*E ,D< +;E ?*EB # ' 4 # / // ' &

' & / / , " # ! E # '

/ # / 4 8 E # ' #

/ / / , " 4

For peer review only

< $ # / / , / / /

" / 1 < ' - C A N@*7B

	/	AEB
- / # # / ?*	?@	
/ # F' &G<br>/	;	;+
# / &<br># ' & / 77	;	!
, # # /<br>' % # '		;?
# / %<br>/ , " 77	@	

7 C / % # - '

' / / % # 4 9 %

' # # 4

77 ?* / ' / # %

/ / ' & % / 4

Incidence of shoulder dislocations in the UK

/ # +! ! (' / / #

' # # 3/ / " A5 B4 0 '

3/ A**EB / & &

' / & & + & ;> &

A?EB4

/ # + ;+! ' # / + ;>

& 2 @@* > * , "

& 4 / / / # / % !4

0 A; EB / // #

For peer review only

/ !+ & A C < (* & B4 0 ' F G

A ?4* (4@ 1 M B & 3 ??E / 4 ' #

/ ## / & D 3 # 0 ' ' % >>(

/ # / % 4

!< 8 / / / # ' & / ' + ;>

& , / / / " / 1 @@* > * 2

, / /			E
		+ ;+!	>>
- 0		(?	;
	5	(+ *	?
/ + > &	*+		*
	!> &	(++	*
	! (> &	! >	?
	(;> &	+ @ *	(
8 & 0 D 3 A1 M B	P ?4*	?>	
	?4* (4@	! !@	>
	*4> @4@	! > >	?
	!>4> !(4@	@	?
	Q!*4>	;+?	*
	0	?	(?
1	1 + +;(		(>
	, 1 ! !	??	>
	3 1	> (	
	0	(+?;	?
1 ,	1 + ?*(		(
	1	!	;
	3 1	??	
	0	? +>?	*
, / & 3 >	(?!(		??
		@*>	+
		* !	!
	Q!	(*+	!
"	0 +>>		(
	# (((		@

For peer review only

	)	(?(	@
		;@	
	9	>;	
	D	+>	(
	/	+ +	>
	,	>>*	
	, *;		@
	9	4(+	@
	9	*@	@
	9 0	(;>	@
	6 1 J **;		!
D 3 # 0 ' ' % >>(AC # ' % B	##	;@>	;
		!(*	(
	!	>>	
	(	;@!	
	' %	!>@	?
	0	+ * *	!@

' / # ' & / ' &

2 % 5 4 ' 1 # ' % ;

& ' 1 % + ;> & 4

D / / & # ' & /

' 2 ' (4 % / /

(>4(' >> >>> ' & A@*E ,D< (>4((>4(B # *4* '

>> >>> ' & A@*E ,D< *4* *4*B4 / / %

+ > & A?>4* ' >> >>> ' & H @*E ,D< ?>4* ?>4+BB

/ / / / A5 !B4 2 ' ' #

/ / % H / / +4(' >> >>> ' &

+ > & / *> &

/ ?4+ ' >> >>> ' & + ;> & A5

For peer review only

B4 D / / # / & '

% / / # 4+> A@*E,D< 4* 4+@B4

For peer review only

(< / / / / # ' & / &

, / / / " / 1 @@* > * 2

' / / &		& 7	D / / 77	@*E ,D777	' / / '	D / /	@*E ,D777	%
-								
0	(?	!> >;(>;?	(>4(	(>4((>4(	0 % 5	4+> 4*	4+@	P>4>>
5	(+ *	@ ;(**@	*4*	*4* *4*				
A& B								
+ >	*+	* (* (?	(?4?	(?4? (?4@	+ > % !>	4 +	4 > 4!	P>4>>
!>	(++	>>+ *?+	!?4?	!?4; !?4?	+ > % ! (>	4>>	4@> 4	P>4>>
! (>	! >	!+ >+	(4(	(4((4*	+ > % (;>	4 > 4	4!	P>4>>
(;>	+ @ *	! > *+	4	4 4	!> % ! (>	4*@ 4*	4++	P>4>>
A B								
+ >	!;	+*! >+	?>4*	?>4* ?>4+	+ > % !>	4 !	4 + 4 @	P>4>>
!>	! *??	* (+! ?!>	+*4;	+*4+ +*4;	+ > % ! (>	4 ?	4>* 4!	P>4>>
! (>	! +	+ +* !(?	!;4>	!+4@ !;4>	+ > % (;>	!4>?	4@ !4 (	P>4>>
(;>	(>;	* +@ ?!?	+4	+4 +4	!> % ! (>	4;? 4+@	4?;	P>4>>
A# B								
+ >	((	*@ !++	+4(	+4! +4(	+ > % !>	4!(4 ?	4*	P>4>>
!>	+;?	* *(;*+	4	4 4 +	> % ! (>	4(4 *	4+>	P>4>>
! (>	;>*	+ >+@ ; (	4+	4+ 4+	+ > % (;>	>4@>	>4? 4>>	>4>*
(;>	?>?	* *>@ ; (	?4	?4 ?4	!> % ! (>	4>+ >4@*	4 ?	>4 @+
A % #								
					+ > % + >	(4@	(4((*4(;	P>4>>
					!> % !>	*4!; (4@*	*4?!	P>4>>
					! (> % ! (>	!4 >	4@(!4(?	P>4>>
					(;> % (;>	4(* 4!?	4*	P>4>>

7 & # / / # ' + ;> & @@* > * , " 4
77 / / ' >> >>> ' & 4
777,D / # / % 4

For peer review only

DISCUSSION

Main findings

' ' / # + ;+! ' + ;> & 2

@@* > * # , " 4 ' %

3 / , " // ' #& &

/ ' 4 0 / // A; EB4

% / / (>4(' > > >>> ' &

4 ' >> >>> ' & 4 / / %

+ > & A?>4* ' >> >>> ' & B4 3' / #

/ / / ?4 ' >> >>> ' &

+ ;> & ' % 4

Comparison with other studies

2 / / / '

% , 2 . / 4[?] [@] D 2 ' 1

' % ; & /

' 1 ; ? & , 2 4 #

/ & # & ' & / /1 & /

5 / ; ? & / ' & / 4 D

& # / # / / /

A?EB / /1 & ' & 4 [?] . 3' ' #

/ / /

1 & / / 4

For peer review only

D / / 2 , # / #

' 3/ ' # + > & A2 < ?>4* ' >> >>>

' & % , < @?4! ' >> >>> ' & B4

/ / & 3' & 2 ' & / /

/ , ' / / 4

& / / 1 # #

/ / ' / # / & ' ' # C & /

& # & ' ' # C &

/ ' & ' 4

/ / / # / &

/ / /

1 4 9 1 / & & / / ##

/ C # # / &4 %

1 C 3 & ' / 1

/ 4 / /

/ / # # /

' ' / ' # C & ' / % / 1

/ / / # # 4 # ' /

' / % ' ' # 2 / / /

& ' ' 4 9 / ' ' ' & % /

& # & / # / # / % / &

D # , 3' / A D, B4 *

For peer review only

Clinical Practice Research Datalink dataset dislocation READ codes used to identify shoulder dislocation patients

Dislocation or subluxation of shoulder	
Dislocation of shoulder NOS	
H/O: dislocated shoulder	
Closed reduction of dislocation of shoulder	!" #
Closed traumatic dislocation of shoulder	
Recurrent dislocation of shoulder – anterior	$ %#
Anterior dislocation of shoulder	
Recurrent joint dislocation, of shoulder region	$ %#
Recurrent subluxation of shoulder – anterior	$ %#
Closed traumatic dislocation shoulder jnt. Anterior (sub-coracoid)	
Closed traumatic dislocation shoulder joint, unspecified	
Closed traumatic subluxation, shoulder	&

For peer review only

Anjali Shah[1], Andrew Judge[1,2], Antonella Delmestri[1], Katherine Edwards[1], Nigel K Arden[1,2], Daniel Prieto-Alhambra[1], Tim A Holt[3], Rafael A Pinedo-Villanueva[1], Sally Hopewell[4], Sarah E Lamb[1], Amar Rangan[1, 5, 6], Andrew J Carr[1,] Gary S Collins[1] and Jonathan L Rees[1]

[1] Nuffield Department of Orthopaedics, Rheumatology, and Musculoskeletal Sciences, University of Oxford. Oxford. UK

[2] Faculty of Medicine, University of Southampton, Southampton. UK

[3] Nuffield Department of Primary Care Health Sciences, University of Oxford, Oxford. UK

[4] Oxford Clinical Trials Research Unit, University of Oxford, Oxford. UK

[5] The James Cook University Hospital, South Tees Hospital NHS Foundation Trust, Middlesborough, UK

[6] Department of Health Sciences, University of York, York. UK

*Correspondence to Dr Anjali Shah

Nuffield Department of Orthopaedics, Rheumatology and Musculoskeletal Sciences,

University of Oxford

Botnar Research Centre,

Windmill Road,

Oxford

OX3 7LD

For peer review only

Email: anjali.shah@ndorms.ox.ac.uk

Tel: 01865 223426

Fax: 01865 227966

Word count: 2,763

Key words: shoulder dislocation, incidence, Clinical Practice Research Datalink, CPRD

For peer review only

!"#$ %#

& ' (

This cohort study evaluates the unknown age- and gender-specific incidence of primary shoulder dislocations in the UK.

"

UK primary care data from the Clinical Practice Research Datalink (CPRD) were used to identify patients aged 16-70 years with a shoulder dislocation during 1995-2015. Coding of primary shoulder dislocations was validated using the CPRD general practitioner (GP) questionnaire service.

)

A cohort of 16,763 shoulder dislocation patients aged 16-70 years during 1995-2015 were identified.

)

Incidence rates per 100,000 person-years and 95% confidence intervals were calculated.

$

Correct coding of shoulder dislocation within CPRD was 89% (95% CI: 83%-95%), and confirmation that the dislocation was a ‘primary’ was 76% (95% CI: 67%-85%). Seventy-two percent of shoulder dislocations occurred in men. The overall incidence rate in males was 40.4 per 100,000 person-years (95% CI: 40.4 to 40.4), and in

For peer review only

females was 15.5 per 100,000 person-years (95% CI: 15.5 to 15.5). The highest incidence was observed in 16-20 year-old males (80.5 per 100,000 person-years; 95% CI: 80.5-80.6). Incidence in women increased with age to a peak of 28.6 per 100,000 person-years among those aged 61-70 years.

%

This is the first time the incidence of shoulder dislocations has been studied using primary care data from a national database, and the first time the results for the United Kingdom have been produced. While most primary dislocations occurred in young men, an unexpected finding was that incidence increased in women aged over 50 years, but not in men. The reasons for this are unknown. Further work is commissioned by the National Institute for Health Research (NIHR) to examine treatments and predictors for recurrent shoulder dislocation.

" : The design of this study was approved by the Independent Scientific Advisory Committee (ISAC 15_260) for the Medicines & Healthcare products Regulatory Agency (MHRA).

"

- This is the first time the incidence of shoulder dislocations has been studied in the UK using a large population-based database from primary care.
- A new finding is that shoulder dislocations in women over the age of 50 years increased to a peak of 28.6 per 100,000 person-years in those aged 61-70 years

For peer review only

For peer review only - http://bmjopen.bmj.com/site/about/guidelines.xhtml

- The coding of shoulder dislocation diagnoses was validated by comparison with medical records, using a GP questionnaire.
- The validation exercise did only include the 16-35 years age group, however there is no reason why coding of shoulder dislocations would be different for patients aged up to 70 years.
- While not all GP practices responded to the questionnaire, those that did and did not respond were similar by deprivation, geography and other demographic characteristics.

For peer review only

#$&+ %# &

Shoulder joint dislocations are the most common joint dislocations seen in hospital Accident and Emergency departments and trauma clinics [1, 2] with 80-97% of traumatic glenohumeral dislocations being anterior. [1-6] Traumatic anterior shoulder dislocation most commonly occurs after traumatic injury in young people and usually results in structural problems such as Bankart lesions [7] and the Hills-Sachs lesion [5]. The joint can remain ‘unstable’ and re-dislocation rates have been reported as being as high as 85% [8] or 92% [9] in young people. Surgery is becoming more common as a treatment, especially in sporting athletes, with many surgeons and patients opting for surgery after only one dislocation.

A Swedish population-based study was conducted by Hovelius. [10] This study while small is well cited in the literature, and observed that 1.7% of the population aged 18-70 years had had a shoulder dislocation. In a 25-year follow-up study of patients aged 12-40 years, recurrent dislocation was more common in younger people with 72% of patients aged 12-22 years suffering another dislocation, which dropped to 27% in those aged 30-40 years. [11] Early studies reported high incidence of shoulder dislocation in military and athletic populations, with young men being at greatest risk. [6, 12-15] In Edinburgh, a study of 252 patients aged 15-35 years suffering a shoulder dislocation identified the most common cause (86%) was playing contact sports. [16] Of these 60% suffered a repeat dislocation and the mean time for recurrent dislocation to occur was 13.3 months.

A number of studies report incidences ranging from 11.2 to 26.2 per 100,000 person-years for shoulder dislocations. [2, 17-21] In 2010, Zachilli & Owens [19] examined the

For peer review only

incidence of traumatic shoulder dislocation in patients of all ages presenting to a random sample of 100 hospital emergency departments across the USA during 2002-2006, as recorded in the National Electronic Injury Surveillance System. Seventy-two per cent of dislocations had occurred in men, and the highest incidence was observed in 20-29 year olds (47.8 per 100,000 person-years; 95% CI: 41.0-54.5). The overall incidence rate in males was 34.9 per 100,000 person-years (95% CI: 30.1 to 39.7), and in females was 13.3 per 100,000 person-years (95% CI: 11.6 to 15.0).

In 2014, Leroux et al. [22] evaluated the incidence of primary anterior shoulder dislocation in patients aged 16-70 years who underwent a closed reduction of the shoulder during April 2002 – September 2010 in Ontario, Canada. Seventy-four per cent of all shoulder dislocations were in men, and the highest incidence was observed in men aged 16-20 years (98.3 per 100,000 person-years). The overall adjusted incidence in men and women were similar to figures reported by Zachilli & Owens. [19]

The incidence of primary anterior shoulder dislocations has not been examined using all patients in a national dataset. The incidence of shoulder dislocations in the UK remains unknown and a large population-based UK study has not been previously undertaken.

The aim of this study was to evaluate the age- and gender-specific incidence of primary shoulder dislocations as recorded in primary care during the 20-year period between 1995 and 2015 in the United Kingdom.

For peer review only

For peer review only

,-#.&+"

+

Population-based primary care data from the Clinical Practice Research Datalink (CPRD) were used to identify a cohort of patients with a shoulder dislocation aged 16-70 years during 1995-2015 in the UK. The design of this study was approved by the Independent Scientific Advisory Committee (ISAC 15_260) for the Medicines & Healthcare products Regulatory Agency (MHRA).

The CPRD covers 11.3 million people from 674 UK general practices, with a current representative coverage of approximately 6.9% of the UK population who are broadly representative of the UK population in terms of age, sex and ethnicity.[23] While the data are anonymised at the patient and general practice level, demographic information at the patient level on age, gender, geographic region, body mass index (BMI, kg/m^2), smoking status (current smoker, ex-smoker, and non-smoker) and drinking status (current drinker, ex-drinker, and non-drinker) were available. Data on the Index of Multiple deprivation 2004 [24] for English patients were linked with the CPRD data. Charlson co-morbidity score was calculated using a list of pre-defined CPRD READ codes. CPRD population denominators for the whole CPRD dataset by individual year, age and gender were also obtained.

)

Shoulder dislocations were identified using READ codes as defined *a priori* after consensus by specialist shoulder clinicians experienced in clinical practice and epidemiological research (Supplementary table A). To ensure that only primary

For peer review only

shoulder dislocations had been captured, patients had to have no recorded shoulder dislocations in the two years of clinical data (a 2-year wash-out period was defined using the date the GP practice was classified as ‘up-to-standard’ and the date the patient first registered at the GP practice) preceding the identified shoulder dislocation. A pre-defined set of exclusion criteria were applied to the CPRD dataset (Figure 1).

/ %)$+

A separate internal validation exercise of the coding of shoulder dislocations within CPRD was conducted among patients aged 16-35 years who were diagnosed during 1 April 1997-31 March 2015 in England using the same READ codes for shoulder dislocations, as well as the same two-year wash-out period. The age range used for this validation exercise was not entirely inclusive of the present study, but tested the same coding used in the older age group. The 16-35 years age range was used to inform both this study and a future commissioned study that will include linked hospital data among patients aged 16-35 years because this group currently have the highest level of priority for clinical interventions.

A GP questionnaire was designed based on a validation algorithm (Supplementary figure B) and with the assistance of GPs. A random sample of 172 patients was selected and CPRD personnel sent the questionnaire to the patient’s GP practice for a clinician to complete by comparing the records on their CPRD system with the patient’s clinical records. Four written reminders were sent every two weeks to the GP practices. Data from the questionnaires were double-entered into a dataset by a

For peer review only

statistician and a project manager, and queries were resolved by an academic orthopaedic shoulder surgeon.

The following validation criteria were stipulated *a priori* for proceeding with shoulder dislocation research studies using CPRD data:

a) A positive predictive value of accurate shoulder dislocation coding within CPRD of at least 75%.
b) A positive predictive value of accurate ‘primary’ or ‘first-time’ shoulder dislocation coding within CPRD of at least 75%.

"

Descriptive statistics were used to summarise the epidemiology of primary shoulder dislocations by demographic factors. Incidence rates by age and gender per 100,000 person-years and incidence rate ratios with 95% confidence intervals and p values were calculated using STATA v14.1.[25]

$-" 0#"

/ 1 %)$+

A random sample of 172 patients aged 16-35 years in England within the CPRD dataset were selected from a total of 6,046. Of these 172, GPs returned the validation questionnaire for 95 (55%) patients. For an additional two patients GPs confirmed that the patient had transferred out of the practice and no medical records or information on the CPRD system were available for them. The demographic characteristics of: (a) the CPRD cohort aged 16-35 years during 1 April 1997 – 31

For peer review only

March 2015 in England (n=6,046), (b) of the 172 patients randomly selected to have a questionnaire sent to their GP for additional information, and (c) responders and (d) non-responders to the GP questionnaire are given in Table 1.

For peer review only

Table 1: Demographic characteristics of shoulder dislocation patients aged 16-35 years recorded within the Clinical Practice Research Datalink dataset during 1 April 1997-31 March 2015 in England, and responders and non-responders to the CPRD GP validation questionnaire

+	2 3	4) 5 3	$ 3	* 3
Cohort size	6,046	172	97	75
Gender				
- Male	4,991 (83%)	137 (80%)	81 (84%)	56 (75%)
- Female	1,055 (17%)	35 (20%)	16 (16%)	19 (25%)
Median age (years) & interquartile range	24 (20-34)	24 (20-29)	24 (20-29)	24 (19-29)
Median BMI (kg/m^2) & interquartile range	24 (22-27)	24 (21-27)	25 (22-28)	23 (21-26)
Median Charlson co-morbidity index & interquartile range	0 (0-0)	0 (0-0)	0 (0-0)	0 (0-0)
Region				
- East Midlands	263 (4%)	0 (0%)	0 (0%)	0 (0%)
- East of England	673 (11%)	21 (12%)	17 (18%)	4 (5%)
- London	695 (12%)	23 (13%)	11 (11%)	12 (16%)
- North East	133 (2%)	4 (2%)	3 (3%)	1 (1%)
- North West	951 (16%)	29 (17%)	13 (13%)	16 (21%)
- South Central	965 (16%)	32 (19%)	17 (18%)	15 (20%)
- South East Coast	702 (12%)	25 (15%)	12 (12%)	13 (17%)
- South West	743 (12%)	17 (10%)	17 (18%)	0 (0%)
- West Midlands	667 (11%)	15 (9%)	7 (7%)	8 (11%)
- Yorkshire & the Humber	254 (4%)	6 (3%)	0 (0%)	6 (8%)
Index of Multiple Deprivation 2004				
- Affluent	1,279 (21%)	53 (31%)	28 (29%)	26 (35%)
- 2	1,077 (18%)	35 (20%)	20 (21%)	15 (20%)
- 3	958 (16%)	24 (14%)	16 (16%)	8 (11%)
- 4	876 (14%)	38 (22%)	19 (20%)	18 (24%)
- Deprived	624 (10%)	22 (13%)	14 (14%)	8 (11%)
- Missing	1,232 (20%)	0 (0%)	0 (0%)	0 (0%)

For peer review only

%

AS analysed the data and drafted the paper. AJ assisted with the analysis and commented on the paper. AD assisted with managing and cleaning the data, and commented on the paper. KE assisted with the literature review, data access, the internal validation study, and commented on the paper. NKA, DPA, AJC, TAH, RPV, SH, SEL, AR, GSC commented on the paper. JR designed the study, oversaw the analysis and revised the paper.

6 1

The authors would like to express their gratitude for the work of Margaret Smith and Clare Bankhead at the Primary Care Unit of the University of Oxford for facilitating access to Clinical Practice Research Datalink data on shoulder dislocations. The authors would also like to express their gratitude for the work of Robin May and Emma Boyle at the Clinical Practice Research Datalink for facilitating the internal validation study using GP questionnaires.

For peer review only

%

AS, AD, KE, AJC, RPV, SH, SL, GSC and JR have no competing interests to declare.

AJ has received a grant from NIHR HS&DR during the conduct of the study, and has received consultancy, lecture fees and honoraria from Servier, UK Renal Registry, Oxford Craniofacial Unit, IDIAP Jordi Gol, Freshfields Bruckhaus Deringer, has held advisory board positions (which involved receipt of fees) from Anthera Pharmaceuticals, INC., and received research sponsorship from ROCHE.

NKA has received honoraria, held advisory board positions (which involved receipt of fees) and received consortium research grants from Merck (honorarium); Roche, Novartis and Bioiberica (grants); Smith & Nephew, Nicox, Flexion Bioventus and Freshfields (personal fees) outside the submitted work.

DPA has received research grants from Amgen and Laboratoires Servier.

TH is a GP in London, and is a GP advisor for, but not employed by, the Clinical Practice Research Datalink (CPRD).

AR has received personal fees from DePuy Ltd., Aviva Health, AXA PPP Healthcare, AXA PPP International, BUPA, Cigna, SecureHealth, Simplyhealth, Vitality Health (Pru Health) and WPA; a grant from JRI Ltd.; and has a patent for a shoulder replacement prosthesis.

For peer review only

- (

This study was approved by the Independent Scientific Advisory Committee (ISAC) for the UK Medicines and Healthcare products Regulatory Agency (MHRA) (ISAC Approval Number 15_260RAR).

$

This project was funded by the National Institute of Health and Research (NIHR) Health Technology Assessment (HTA) Programme (project number: 14/160). The views and opinions expressed therein are those of the authors and do not

necessarily reflect those of the HTA programme, NIHR, NHS or the Department of Health. All researchers are independent from the study funders. This study is based in part on data from the Clinical Practice Research Datalink obtained under licence from the UK Medicines and Healthcare products Regulatory Agency. The funding source had no role in the design and conduct of the study, in the collection, analysis and interpretation of the data, or in the preparation, review or approval of the manuscript.

For peer review only

7

Figure 1: Shoulder dislocation exclusion flow chart for patients aged 16-70 years during 1995-2015 within the Clinical Practice Research Datalink, UK.

Figure 2: Percentage of primary shoulder dislocation patients by age (16-70 years) and gender recorded within the Clinical Practice Research Datalink during 1995–2015, UK. Blue bars represent male patients, and red bars represent female patients.

Legend: 95% confidence intervals are included in this figure, but they are so close to the main data points that they cannot be seen.

Figure 3: Shoulder dislocation incidence rates by age and gender during 1995-2015 in the UK, using data from the Clinical Practice Research Datalink.

For peer review only

$

1. Cutts S, Prempeh M, Drew S. Anterior shoulder dislocation. Ann R Coll Surg Engl. 2009;91(1):2-7.

2. Kroner K, Lind T, Jensen J. The epidemiology of shoulder dislocations. Arch Orthop Trauma Surg. 1989;108(5):288-90.

3. Pope EJ, Ward JP, Rokito AS. Anterior shoulder instability - a history of arthroscopic treatment. Bull NYU Hosp Jt Dis. 2011;69(1):44-9.

4. Goss TP. Anterior glenohumeral instability. Orthopedics. 1988;11(1):87-95.

5. Hill HA & Sachs MD. The grooved defect of the humeral head. Radiology. 1940;35:690-700.

6. Owens BD, Dawson L, Burks R, et al. Incidence of shoulder dislocation in the United States military: demographic considerations from a high-risk population. J Bone Joint Surg Am. 2009;91(4):791-6.

7. Bankart AS. Recurrent or Habitual Dislocation of the Shoulder-Joint. Br Med J. 1923;2(3285):1132-3.

8. Henry JH, Genung JA. Natural history of glenohumeral dislocation--revisited. Am J Sports Med. 1982;10(3):135-7.

9. Rowe CR. Prognosis in dislocations of the shoulder. J Bone Joint Surg Am. 1956;38-A(5):957-77.

10. Hovelius L. Incidence of shoulder dislocation in Sweden. Clin Orthop Relat Res. 1982(166):127-31.

For peer review only

11. Hovelius L, Olofsson A, Sandstrom B, et al. Nonoperative treatment of primary anterior shoulder dislocation in patients forty years of age and younger. a prospective twenty-five-year follow-up. J Bone Joint Surg Am. 2008;90(5):945-52.

12. Owens BD, Agel J, Mountcastle SB, et al. Incidence of glenohumeral instability in collegiate athletics. Am J Sports Med. 2009;37(9):1750-4.

13. Owens BD, Duffey ML, Nelson BJ, et al. The incidence and characteristics of shoulder instability at the United States Military Academy. Am J Sports Med. 2007;35(7):1168-73.

14. Milgrom C, Mann G, Finestone A. A prevalence study of recurrent shoulder dislocations in young adults. J Shoulder Elbow Surg. 1998;7(6):621-4.

15. Rhee YG, Cho NS, Cho SH. Traumatic anterior dislocation of the shoulder: factors affecting the progress of the traumatic anterior dislocation. Clin Orthop Surg. 2009;1(4):188-93.

16. Robinson CM, Howes J, Murdoch H, et al. Functional outcome and risk of recurrent instability after primary traumatic anterior shoulder dislocation in young patients. J Bone Joint Surg Am. 2006;88(11):2326-36.

17. Simonet WT, Melton LJ, 3rd, Cofield RH, et al. Incidence of anterior shoulder dislocation in Olmsted County, Minnesota. Clin Orthop Relat Res. 1984(186):186-91.

18. Nordqvist A, Petersson CJ. Incidence and causes of shoulder girdle injuries in an urban population. J Shoulder Elbow Surg. 1995;4(2):107-12.

19. Zacchilli MA, Owens BD. Epidemiology of shoulder dislocations presenting to emergency departments in the United States. J Bone Joint Surg Am. 2010;92(3):542-9.

20. Liavaag S, Svenningsen S, Reikeras O, et al. The epidemiology of shoulder dislocations in Oslo. Scand J Med Sci Sports. 2011;21(6):e334-40.

For peer review only

21. Yang NP, Chen HC, Phan DV, et al. Epidemiological survey of orthopedic joint dislocations based on nationwide insurance data in Taiwan, 2000-2005. BMC Musculoskelet Disord. 2011;12:253.

22. Leroux T, Wasserstein D, Veillette C, et al. Epidemiology of primary anterior shoulder dislocation requiring closed reduction in Ontario, Canada. Am J Sports Med. 2014;42(2):442-50.

23. Herrett E, Gallagher AM, Bhaskaran K, et al. Data Resource Profile: Clinical Practice Research Datalink (CPRD). Int J Epidemiol. 2015;44(3):827-36.

24. Neighbourhood Renewal Unit - Office of the Deputy Prime Minister. The English Indices of Deprivation 2004 (revised). London: Neighbourhood Renewal Unit, Office for the Deputy Prime Minister; 2004.

25. Statacorp. Stata Statistical Software: release 14. College Station, TX: Statcorp LP. 2015.

26. National Institute for Health and Care Excellence. Falls in older people: assessing risk and prevention (CG161). London 2013.

27. Van Staa TP, Abenhaim L, Cooper C, et al. The use of a large pharmacoepidemiological database to study exposure to oral corticosteroids and risk of fractures: validation of study population and results. Pharmacoepidemiol Drug Saf. 2000;9(5):359-66.

28. Thomas SL, Edwards CJ, Smeeth L, et al. How accurate are diagnoses for rheumatoid arthritis and juvenile idiopathic arthritis in the general practice research database? Arthritis Rheum. 2008;59(9):1314-21.

For peer review only

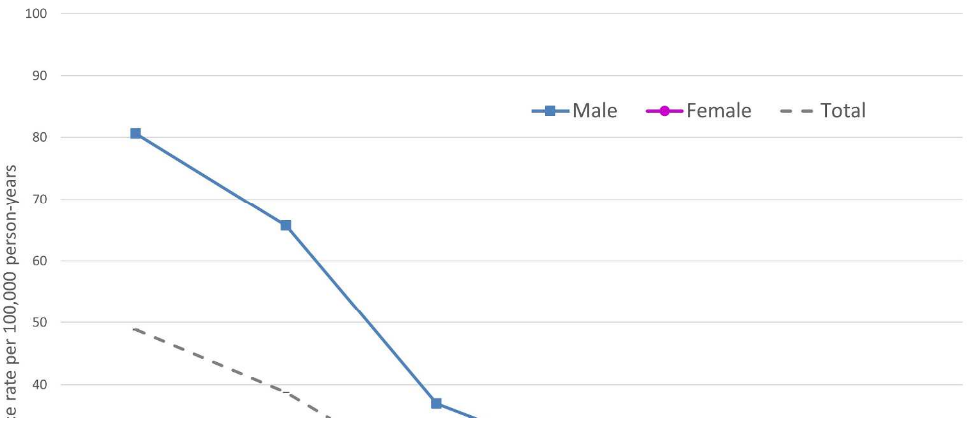

Figure 3: Shoulder dislocation incidence rates by age and gender during 1995-2015 in the UK, using data from the Clinical Practice Research Datalink. Legend: 95% confidence intervals are included in this figure, but they are so close to the main data points that they cannot be seen.

254x190mm (300 x 300 DPI)

y only

Validation Algorithm

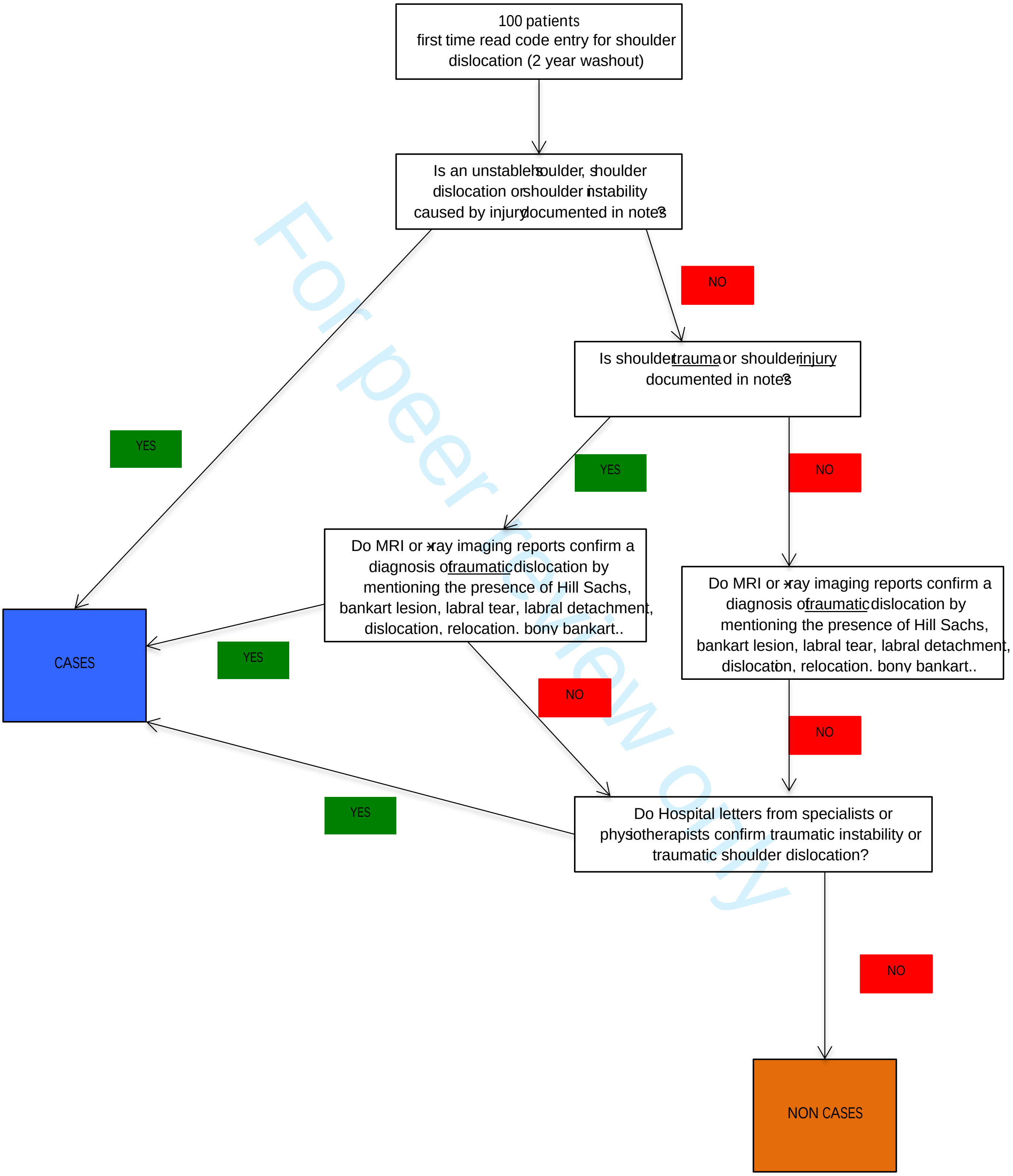

For peer review only

STROBE Statement—checkl[redacted]

	Item No	
**Title and abstract** 'Cohort' page 1 and page 3	1	[redacted]
**Introduction**		
Background/rationale page 6	2	[redacted]
Objectives page 7	3	[redacted]
**Methods**		
Study design page 9	4	[redacted]
Setting pages 9-10	5	[redacted]
Participants pages 9-10	6 N/A	[redacted]
Variables page 9	7	[redacted]
Data sources/ measurement page 9	8*	[redacted]
Bias pages 10-11	9	[redacted]
Study size page 31	10	[redacted]
Quantitative variables page 9	11	[redacted]
Statistical methods page 11 Only descriptive statistics and incidence rates. No multivariate analysis or follow-up needed.	12 N/A N/A N/A N/A	[redacted]

Continued on next page

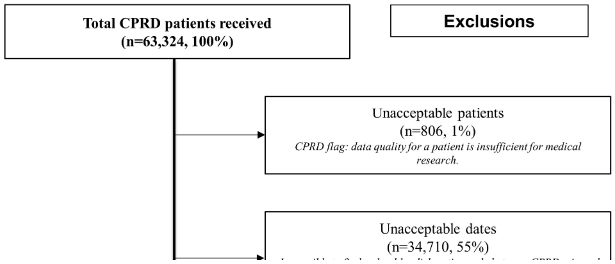

Figure 1: Shoulder dislocation exclusion flow chart for patients aged 16-70 years during 1995-2015 within the Clinical Practice Research Datalink, UK.

190x338mm (300 x 300 DPI)

Validation Algorithm

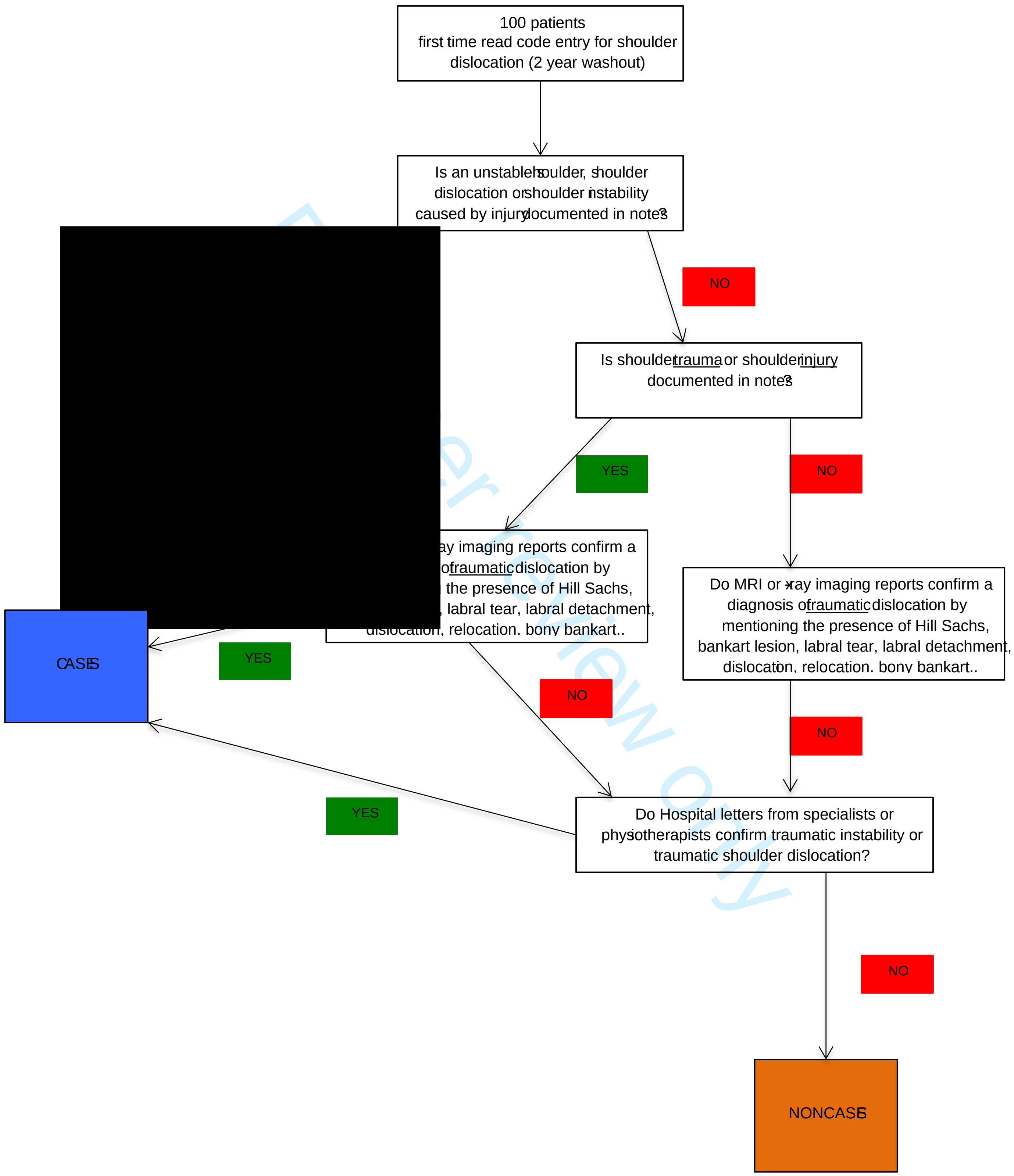

For peer review only

STROBE Statement—checklist of items that shou [illegible]

	Item No	
**Title and abstract** 'Cohort' page 1 and page 3	1	(*a*) Indicate the study's [illegible]
		(*b*) Provide in the abstr[illegible] and what was found
**Introduction**		
Background/rationale page 6	2	Explain the scientific b[illegible]
Objectives page 7	3	State specific objective[illegible]
**Methods**		
Study design page 9	4	Present key elements o[illegible]
Setting pages 9–10	5	Describe the setting, lo[illegible] exposure, follow-up, an[illegible]
Participants pages 9-10	6	(*a*) <u>*Cohort study*</u>—Give [illegible] selection of participants [illegible]
		Case-control study—G[illegible] case ascertainment and [illegible] and controls
		Cross-sectional study—[illegible] selection of participants [illegible]
	N/A	(*b*) *Cohort study*—For [illegible] exposed and unexposed [illegible]
		Case-control study—F[illegible] controls per case
Variables page 9	7	Clearly define all outco[illegible] modifiers. Give diagno[illegible]
Data sources/ measurement page 9	8*	For each variable of in[illegible] assessment (measureme[illegible] is more than one group [illegible]
Bias pages 10-11	9	Describe any efforts to [illegible]
Study size page 31	10	Explain how the study [illegible]
Quantitative variables page 9	11	Explain how quantitativ[illegible] describe which grouping[illegible]
Statistical methods page 11 Only descriptive statistics and incidence rates. No multivariate analysis or follow-up needed.	12	(*a*) Describe all statistica[illegible]
	N/A	(*b*) Describe any method[illegible]
	N/A	(*c*) Explain how missing [illegible]
	N/A	(*d*) *Cohort study*—If app[illegible]
		Case-control study—If a[illegible] addressed
		Cross-sectional study—[illegible] sampling strategy
	N/A	(*e*) Describe any sensitiv[illegible]

Continued on next page

For peer review only - http://bmjopen.bmj.com/site/about/guidelines.xhtml

**Results**		
Participants pages 11, 15 and 31 Figure 1.	13*	[illegible]
Descriptive data pages 11-17	14*	N [illegible]
Outcome data pages 17-19	15*	[illegible]
Main results pages 11-19 pages 32-33 for Figures	16	[illegible]
Other analyses	17	N/[illegible]
**Discussion**		
Key results page 20	18	[illegible]
Limitations page 22	19	[illegible]
Interpretation pages 20-21	20	[illegible]
Generalisability pages 20-21	21	[illegible]
**Other information**		
Funding pages 25-26	22	[illegible]

*Give information sepa[illegible] unexposed groups in co[illegible]

Note: An Explanation a[illegible] published examples of t[illegible] available on the Web si[illegible] http://www.annals.org/[illegible] available at www.strob[illegible]

For peer review only - http://bmjopen.bmj.com/site/about/guidelines.xhtml
